# Supplementary figures and images for: Mapping the Oncological Basis Dataset to the Standardized Vocabularies of a Common Data Model: A Feasibility Study
Source: Cancers (Basel). 2023 Aug 11;15(16):4059. doi: 10.3390/cancers15164059 (PMC10452256; doi:10.3390/cancers15164059)

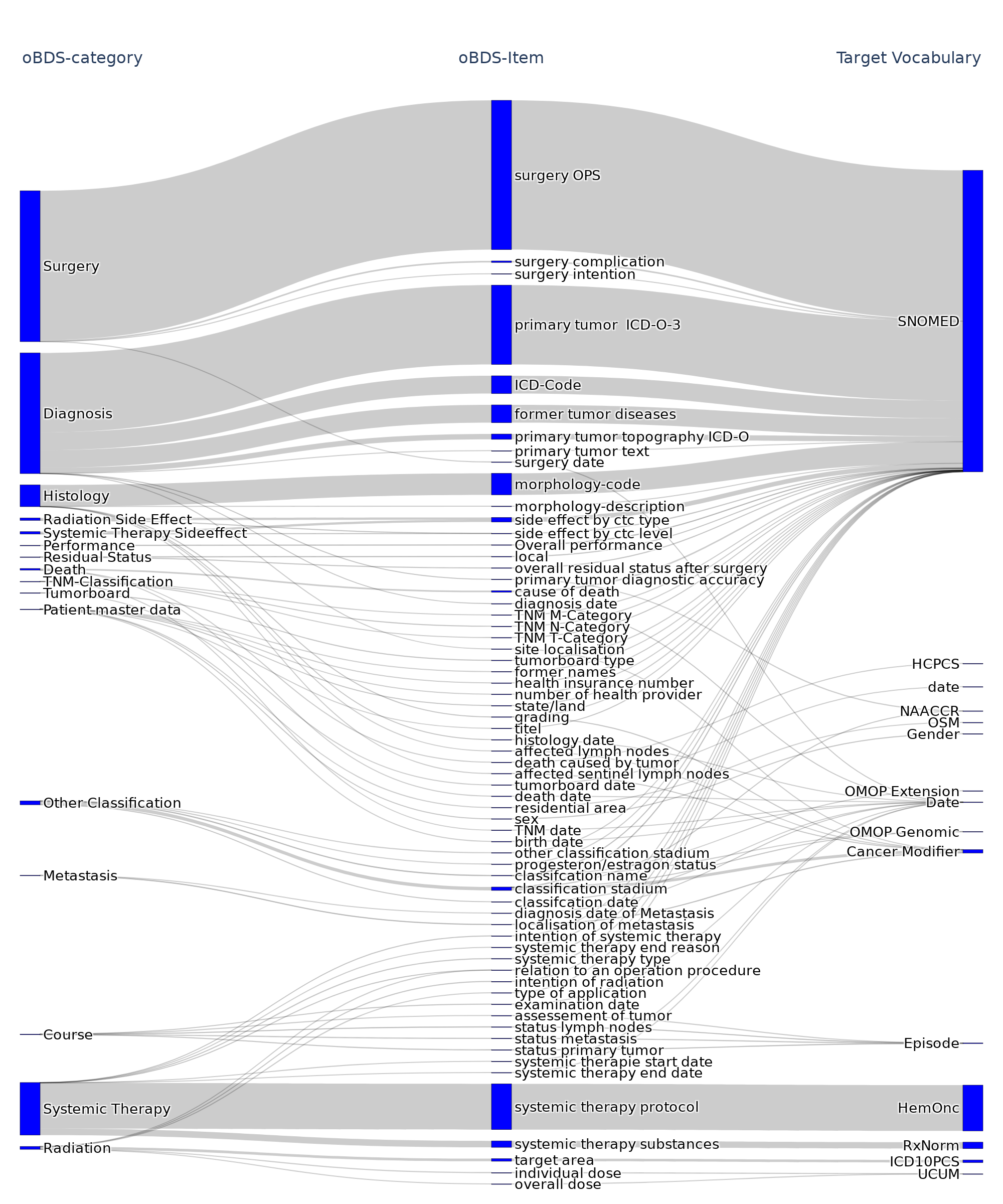

Supplement: Supplementary file 1 [file cancers-15-04059-s001.zip › Sup_Fig_1.PNG]

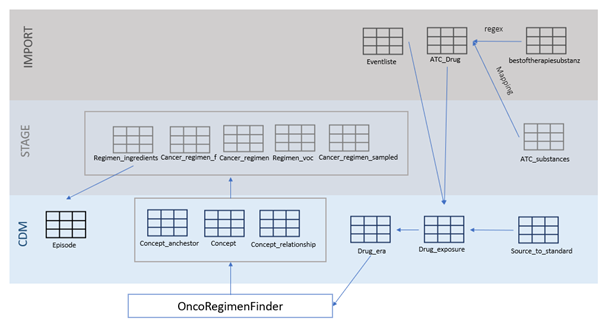

Supplement: Supplementary file 1 [file cancers-15-04059-s001.zip › Sup_Fig_2.png]
